# Study of Benzofuroquinolinium Derivatives as a New Class of Potent Antibacterial Agent and the Mode of Inhibition Targeting FtsZ

**DOI:** 10.3389/fmicb.2018.01937

**Published:** 2018-08-17

**Authors:** Yuan-Yuan Zheng, Ruo-Lan Du, Sen-Yuan Cai, Zhi-Hua Liu, Zhi-Yuan Fang, Ting Liu, Lok-Yan So, Yu-Jing Lu, Ning Sun, Kwok-Yin Wong

**Affiliations:** ^1^Institute of Natural Medicine & Green Chemistry, School of Chemical Engineering and Light Industry, Guangdong University of Technology, Guangzhou, China; ^2^Department of Applied Biology and Chemical Technology and the State Key Laboratory of Chirosciences, The Hong Kong Polytechnic University, Kowloon, Hong Kong; ^3^The Fifth Affiliated Hospital of Guangzhou Medical University, Guangzhou, China; ^4^Goldenpomelo Biotechnology Co., Ltd., Meizhou, China; ^5^Guangdong Provincial Key Laboratory of New Drug Screening, Guangzhou, China

**Keywords:** bacterial resistance, antibacterial activity, cell division, FtsZ inhibitor, FtsZ polymerization

## Abstract

New generation of antibacterial agents are urgently needed in order to fight the emergence of multidrug-resistant bacteria. FtsZ is currently identified as a promising target for new types of antimicrobial compounds development because of its conservative characteristics and its essential role played in bacterial cell division. In the present study, the antibacterial activity of a series of benzofuroquinolinium derivatives was investigated. The results show that the compounds possess potent antibacterial activity against drug resistant pathogens including MRSA, VREF and NDM-1 *Escherichia coli*. Biological studies reveal that the compound is an effective inhibitor that is able to suppress FtsZ polymerization and GTPase activity and thus stopping the cell division and causing cell death. More importantly, this series of compounds shows low cytotoxicity on mammalian cells and therefore they could be new chemotypes for the development of new antibacterial agents targeting the cell-division protein FtsZ.

## Introduction

The use of antibiotics to treat infectious diseases is a common and very effective practice in the past decades (Brown, 2015). However, because of our past misuse and abuse of antibiotics, multi-drug resistant bacteria, such as methicillin-resistant *Staphylococcus aureus* (MRSA) and vancomycin-resistant *Enterococcus faecium* (VREF), pose the greatest threat to human health (Levy and Marshall, 2004; Fischbach and Walsh, 2009; Willyard, 2017). The World Health Organization (WHO) reported that antibacterial resistance is one of the biggest health problems and the world is running out of antibiotics (WHO, 2017). Bacteria can be intrinsically resistant to antibiotics or develop resistance rapidly after exposure to antibiotics (Blair et al., 2015). The mechanisms of antibiotic resistance are generally developed through the following pathways: (i) bacteria producing specific enzyme to inactivate antibacterial drugs (Lin et al., 2015); (ii) modifying drug targets to render antibiotics ineffective (Walsh, 2003; Mi et al., 2016); (iii) changing the permeability of the outer membrane to prevent drugs entering the cell body, affecting the efflux system to pump out the drugs (Walsh, 2003; Nikaido and Pagès, 2012; Wang et al., 2018). Therefore, new generation of antibacterial agents that act through novel mechanisms and/or on novel targets are needed (Payne, 2008; Rice, 2008).

It has been recognized that new drug targets of antibacterial agents is possibly obtained through the study of bacterial cell division machinery (Margalit et al., 2004). While in the bacterial cell-division cycle, filamenting temperature-sensitive mutant Z (FtsZ) is targeted as one of the most important proteins. Because it undergoes GTP-dependent polymerization at mid-cell and recruits the other cell division proteins to complete the division process (Bi and Lutkenhaus, 1991; Lutkenhaus, 1993). In addition, the characteristics of FtsZ proteins are conserved in most bacteria (Lock and Harry, 2008; Kapoor and Panda, 2009).

**FIGURE 1:**
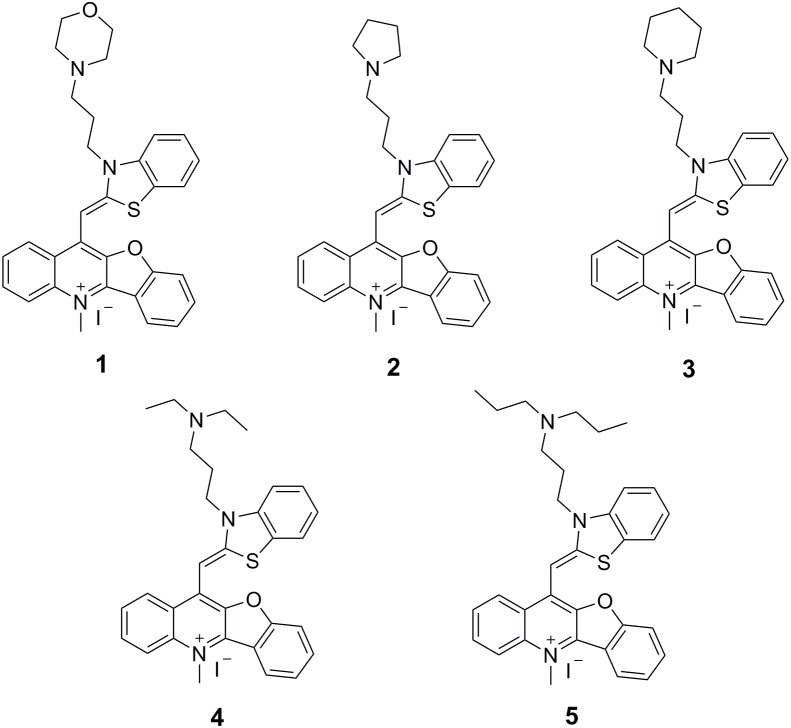
Chemical structures of benzothiazole-substituted benzofuroquinolinium derivatives **1**–**5**.

Over the past few years, some FtsZ-targeting compounds were reported to inhibit bacterial cell division through disrupting GTPase activity and polymerization of FtsZ (Domadia et al., 2007; Haydon et al., 2008; Ruiz-Avila et al., 2013; Stokes et al., 2014; Haranahalli et al., 2016; Liu et al., 2018). These studies suggested that the molecules impair bacterial growth through disrupting the dynamic assembly and GTP hydrolysis of FtsZ. These inhibitors can be divided into natural products and chemically synthesized small molecules. Natural products mainly include curcumin, berberine, totarol and cinnamaldehyde (Domadia et al., 2007; Jaiswal et al., 2007; Domadia et al., 2008; Kaur et al., 2010). The synthesized small molecules mainly include 3-MBA derivatives, rhodanine derivatives and taxane derivatives (Haydon et al., 2008; Singh et al., 2012, 2014; Bi et al., 2018). Recently, we successfully screened some FtsZ inhibitors with strong antibacterial effects including berberine derivatives (Sun et al., 2014), quindolinium derivatives (Sun et al., 2017a), pyrimidine derivatives (Chan et al., 2017), and tiplaxtinin (Sun et al., 2017c), and found that molecular structures bearing a benzothiazole or quinolinium scaffold is commonly able to inhibit the bacterial cell division and disrupt FtsZ function. In this study, on the basis of our understanding on the benzothiazole and quinolinium molecular frameworks, we attempted to further study benzothiazole-substituted benzofuroquinolinium derivatives **1–5** (**Figure 1**) to search for FstZ inhibitors with potent antimicrobial activity. These compounds were published as G-quadruplex fluorescent probes (Lu et al., 2015), but their binding affinities were low. However, our previous reports about FtsZ-targeting quinolinium derivatives (Sun et al., 2017a, 2018) encourage us to investigate the antibacterial activities and their mode of action of these benzofuroquinolinium derivatives **1–5**. The results obtained reveal that these derivatives can effectively inhibit the GTPase activity and polymerization of FtsZ. The compounds also exhibit cell division inhibition phenomenon with a promising antibacterial activity against bacterial strains including the drug-resistant strains.

## Materials and Methods

### Determination of Minimum Inhibitory Concentration

The minimum inhibitory concentrations (MICs) of the test compounds were determined using a broth microdilution method according to the Clinical and Laboratory Standards Institute (CLSI) guidelines (Wikler et al., 2009). A single colony of test bacterial strain was inoculated from TSB agar plate to 5 mL Mueller Hinton broth (MHB) or Cation-adjusted Mueller hinton broth (CA-MHB) and then overnight incubated at 37°C. Cells were diluted to a final concentration of approximately 5 × 10^5^ CFU/mL in a 96-well microtiter plate. The compounds were then added at a series concentration (0.125, 0.25, 0.5, 1, 2, 4, 8, 16, 32, 64 μg/mL) and the plate was incubated at 37°C for 18 h. The MICs are determined as the minimum concentration of the visually clear wells. 10 μl of broth cultures from the MIC assays were incubated overnight on the MH agar at 37°C. The minimum bactericidal concentration (MBC) was reported as the lowest concentration at which colonies were not observed (Wikler et al., 2009). Three independent assays were performed for all the tests.

### Time Killing Curve

*Staphylococcus aureus* ATCC 29213 and *Escherichia coli* ATCC 25922 were diluted to approximately 1 × 10^5^ CFU.mL^-1^ with MHB, various concentrations of compounds were added, and the cultures were incubated at 37°C (Wikler et al., 2009; Sun et al., 2017a). Hundred microliter samples were removed and diluted appropriate times and then spread on to MH agar at 1, 2, 4, 6, 8, 24 h. Agar plates were incubated at 37°C for 24 h to count the cell colonies (CFU.ml^-1^). Three independent assays were performed for all the tests.

### Synergistic Effect With Methicillin

Synergistic effect of these compounds with methicillin against *S. aureus* ATCC BAA41 (MRSA) was tested using a checkerboard method by following the previous reported procedures (Pillai et al., 2005). In addition, we also detected the growing curve of *S. aureus* ATCC BAA41 cultured with or without our compounds with methicillin. Cells were cultured overnight at LB broth and then diluted to 1 × 10^5^ CFU/mL with CA-MHB (Liu et al., 2000; Wang et al., 2017). Compounds and methicillin at a different combination concentration were added and cells were then incubated at 37°C. OD_600_ of the mixtures were measured using a UV-visible spectrophotometer at 1, 2, 4, 6, 8, 24 h. FIC index = MICA/A + MICB/B, A and B stand for MIC when acting alone. FIC index: <0.5, two drugs for synergy; 0.5–1 for the additive effect; 1–2 is irrelevant; >2 for the antagonistic effect. Three independent assays were performed for all the tests.

### Bacterial Morphology and Membrane Staining Studies

The *B. subtilis* 168 cells or *E. coli* 25922 were overnight incubated at 37°C in 5 mL Luria-Bertani (LB) broth (Sun et al., 2017b). Then the cells were diluted to 1 × 10^5^ CFU/mL, treated with different concentrations of compounds at 37°C for 4 h. For membrane staining, the *B. subtilis* cells were further incubated with 1.6 μM of FM 4–64 for an additional 30 min at 37°C without shaking before harvested. The bacterial cells were then harvested and re-suspended in PBS containing 0.25% agarose. Then the sample mixtures were placed on a microscopic slide pre-treated with 0.1% (w/v) poly-L-lysine. The morphology of the bacterial cells was observed using an Olympus FSX100 microscope at 400× magnification.

### Visualization of Z-ring in Bacterial Cells

The assay using a strain of *B. subtilis* 168 containing an IPTG-inducible plasmid, it can produce the green fluorescence protein (GFP)-tagged FtsZ to make the Z-ring visible (Sun et al., 2014). A Single colonies of the *B. subtilis* was overnight incubated at 37°C in 5 mL LB broth, and then were diluted to 1 × 10^5^ CFU/mL with MHB containing 40 μM IPTG (Isopropyl β-D-1-thiogalactopyranoside). The cells were treated with the test compounds at the MIC concentration for 4 h at 37°C. After that they were centrifuged and re-suspended in PBS and then observed using the Nikon N-SIM Super-resolution Imaging System.

### GTPase Activity Assay

*Escherichia coli* FtsZ was cloned, overexpressed, and purified as previous report (Li and Ma, 2015). The GTPase activity of *E. coli* FtsZ was measured by using an ATPase/GTPase Activity Assay Kit (SIGMA MAK113) according to an optimized protocol and the Kit instructions (Sun et al., 2014). The *E. coli* FtsZ protein was diluted at a final concentration of 10 μm in MOPS-KOH (50 mM MOPS, 50 mM KCl, 5 mM MgCl_2_, pH 6.5). The Assay Reaction Mixes contains of 20 μL Assay Buffer, 5 μL protein and 5 μL DMSO/compounds (0.5, 1, 2, 4, 8, 16, 32, 64 μg/mL). Mix Assay Buffer with 4 mM GTP in a 2:1 ratio to set up the Reaction Mix. Add 30 μL of Reaction Mix to each sample, background blank, and negative control well. Incubate the reaction for 30 min at room temperature. Then, 200 μL of reagents were added to each well and the plates were incubated for an additional 30 min at room temperature to terminate the enzyme reaction and generate the colorimetric product. The absorbance was read at 620nm for all samples, standards, blanks, and controls. The phosphorus standard curve has been measured and shown at **Supplementary Table S2**, the standard curve equation is *y* = 0.0092x + 0.289 and *R*^2^ = 0.996. Each assay was carried out in triplicates.

### Polymerization Assay

*Escherichia coli* FtsZ protein was diluted at a final concentration of 10 μm in MOPS-KOH (50 mM MOPS, 50 mM KCl, 5 mM MgCl_2_, pH 6.5). Compound **5** was added to the protein at a series of concentrations (8, 16, 32, 64 μg/mL) and DMSO was used as negative control. Then the reaction was initiated by adding 1mM GTP and the mixtures were incubated at 25°C for 1 h. Then the samples were centrifuged at 14,000x rpm for 60 min, pellets were re-suspended in MOPS-KOH and then analyzed by 12% SDS-PAGE gel. Gels were stained with Instant Blue and the protein content of binding bands were measured by densitometric quantification using Image J software (Mukherjee et al., 2001; Margalit et al., 2004; Andreu et al., 2010). Each assay was carried out in triplicates.

### Drug Resistance Study

The drug resistance study of compound **1**–**5** was performed by following the protocol of previous study (Lin et al., 2017). The initial MIC values of Compound **1–5** and methicillin against *S. aureus* ATCC 29213 were determined according to method described above. The bacteria in the 0.5× MIC wells from the above experiment were diluted to approximately 1× 10^5^ CFU.mL^-1^ with MHB for the next MIC test. Various concentrations of compounds were added to the corresponding bacteria, the MICs are determined after incubated 24 h at 37°C. The process was repeated continuously for 25 days, assays were carried out with biological replicates.

### Molecular Modeling

Molecular modeling was carried out by CDOCKER of Discovery Studio 2016, the X-ray crystal structure of the *S. aureus* FtsZ bound with PC190723 (PDB code: 4DXD) and GDP was downloaded from the RCSB Protein Data Bank^1^. The ligands and water molecules were removed, and the protein was prepared for docking using an automated protein preparation protocol.

### Cytotoxicity

Compounds **1**–**5** were tested with Mouse fibroblasts cells (L929), renal epithelial cells (HK-2) and human hepatocellular liver carcinoma cells (HepG2) by MTT method to determine the cytotoxicity of eukaryotic mammalian cells. Cells are resuscitated to log phase, then collected and diluted with cell culture medium to about 8000 cells per mL. For the MTT assay, cells were 100 μL per well seeded in a 96-wells microplate and incubated at 37°C about 16 h until cells adhere at the bottom of wells. Then cells were treated with compounds for 24 h, 3-(4,5-dimethyl-2-thiazolyl)-2,5-diphenyl-2-H-tetrazolium bromide (MTT) were added into cells at a final concentration of 0.5 mg/mL for another 4 h to form formazan, then the formazan were dissolved with DMSO and the concentration was measured by reading OD_490_ with a multi-function microporous plate detector (Sun et al., 2017b). Each assay was carried out in triplicates.

### Hemolytic Activity

Compounds **1**–**5** were tested with Human erythrocytes (Hongquan bio-tel, GZ) at a concentration of 2% as previous report described (Zheng et al., 2017). The assay volumes are 200 μL. Different concentrations of compounds were added 100 μL per well to the 96-well plate, then each well added 100 μL of human blood. After incubated 1 h at 37°C, the plate was centrifuged at 500 × *g* for 5 min, then 100 μL of the supernatant were to determine the absorbance at 540 nm. Each assay was carried out in triplicates.

### Synthesis of Benzothiazole-Substituted Benzofuroquinolinium Derivatives

The test compounds were synthesized according to the previous reported procedures (Lu et al., 2015). Their structures and purity were confirmed by NMR and ESI-MS (refer to **Supplementary Materials**).

## Results

### Minimum Inhibitory Concentration of Compounds 1–5

The antibacterial activity of the compounds was evaluated, and the results show that all compounds possess potent antibacterial activity against the tested strains (**Table 1**). For Gram-positive bacteria, the compounds inhibited the growth of antibiotic-susceptible *S. aureus* and methicillin-resistant *S. aureus* (MRSA) with minimum inhibitory concentrations (MIC) in the range of 0.25–8.0 μg/mL (0.48–16.17 μM). Among the compounds, **5** containing an extended *N*-isopropyl-*N*-propylpropan-1-amine group from the benzothiazole moiety was the most effective one against MRSA with MIC values ranged from 0.5–1.0 μg/mL (0.96–1.91 μM). Its antibacterial activity is over 200-fold better than methicillin. In addition, the growth of vancomycin-resistant *E. faecium* (VRE) was inhibited by **5** with an MIC value of 1 μg/mL (1.91 μM). The antibacterial effects of **5** on the VRE was much more potent than that of vancomycin (MIC > 64 μg/mL) (Sun et al., 2017a). For Gram-negative bacteria, the compounds can inhibit the growth of *E. coli* and its antibiotics resistant mutant with MIC values of 1–8 μg/mL (1.91–15.73 μM). When tested with NDM-1 *E. coli*, methicillin shows just little effect even at the concentration MIC > 64 μg/mL (>159.05 μM). It is noteworthy that **5** can effectively inhibit NDM-1 producing *E. coli* with a much lower MIC value of 1 μg/mL (1.91 μM). In addition, *Acinetobacter baumannii*, *Pseudomonas aeruginosa*, and *Klebsiella pneumoniae*, which are resistant to most of the clinical antibiotics can be inhibited by our compounds with MIC values of 4–64 μg/mL (7.65–125.82 μM). Compound **5** can act effectively against these multi-drug resistant Gram-negative strains with MIC values of 4–8 μg/mL (7.65–15.30 μM) (**Table 1**). The minimum bactericidal concentration (MBC) of **5** is 1 μg/mL (1.91 μM) against *E. coli* ATCC 25922 and *S. aureus* ATCC 29213, suggesting that the antibacterial activity of our compounds with the same mode of bactericidal effect.

**Table 1:** Minimum inhibitory concentrations of compounds **1–5** against a series of bacterial strains.

Organism	MIC (μg/mL)					
	
	1	2	3	4	5	Methicillin
(+)*B. subtilis* 168	0.5	1	0.5	2	0.25	<1
(+)*S. aureus* ATCC 29213	4	4	4	4	0.25	<1
(+)*S. aureus* ATCC 25923	2	2	2	4	1	< 1
(+)*S. aureus* ATCC 43300^a^	4	4	4	4	1	512
(+)*S. aureus* ATCC BAA41^a^	1	2	1	2	1	1024
(+)*S. aureus* ATCC 33591^a^	8	4	4	8	1	1024
(+)*S. aureus* ATCC 33592^a^	4	4	2	4	0.5	512
(+)*S. aureus* ATCC BAA-1720^a^	2	4	2	8	1	1024
(+)*E. faecalis* ATCC 29212	16	8	8	16	4	1
(+)*E. faecalis* ATCC 700221	4	8	nd	8	1	1.5
(-)*E. coli* ATCC 25922	8	4	2	4	1	3
(-)*E. coli* ATCC BAA-2469	8	4	2	4	1	>64
(-)*A. baumannii* ATCC 19606	32	16	16	32	8	>64
(-)*P. aeruginosa* ATCC BAA-2108	16	16	16	16	4	>64
(-)*K. pneumoniae* ATCC BAA-2470	64	16	16	32	8	>64


*^*a*^These strains are MRSA. (+) for Gram-positive and (-) for Gram-negative. nd for not detected.*

### Time-Killing Curve Determinations

**FIGURE 2:**
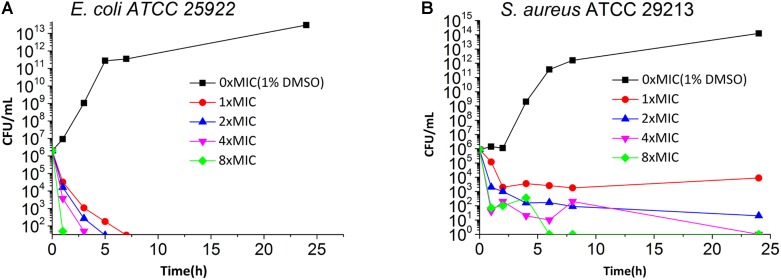
Time Killing Curve of **5** against *E. coli* ATCC 25922 **(A)** and *S. aureus* ATCC 29213 **(B)**. The different concentration of **5** was represented by different colors, 0× MIC (1% DMSO) (black), 1× MIC (red), 2× MIC (blue), 4× MIC (pink) and 8× MIC (green).

Time killing curves were determined by counting bacterial colonies at different time points to determine whether the compounds were bactericidal or not. The results of time killing curves from **5** against *E. coli* ATCC 25922 and *S. aureus* ATCC 29213 are shown in **Figure 2**. The results show that the growth of *E. coli* ATCC 25922 and *S. aureus* ATCC 29213 can be completely inhibited at 1x MIC and at 4x MIC respectively.

### Synergistic Effect of Compound 5 and Methicillin Against MRSA BAA-41

**FIGURE 3:**
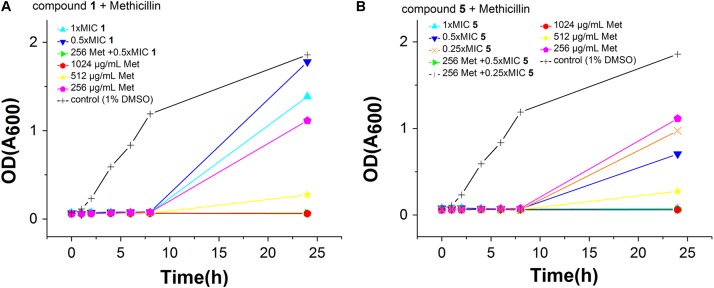
Effects of Compound **2 (A)** and **5** **(B)** combination with methicillin against *S. aureus* ATCC BAA41. The different concentration of compounds was represented by different colors, control (1% DMSO) (black), 1× MIC (cyan), 0.5× MIC (blue) and 0.25× MIC (orange). The different concentration of methicillin was represented by different colors, 1024 μg/mL (red), 512 μg/mL (yellow), and 256 μg/mL (pink). In addition, green and dark gray represent the effect of methicillin with 0.5 or 0.25× MIC of compound, respectively.

To observe whether the compounds can restore the antibacterial activity of methicillin, an MRSA strain (*S. aureus* ATCC BAA41) was treated with methicillin combined with compounds **1**–**5**. The MIC of methicillin against BAA41 is 1024 μg/mL (2544.75 μM), but in the combination test with **5** at the concentration of 25% MIC (0.25 μg/mL) value or with other compounds at the concentration of 50% MIC value, it can be reduced to 256 μg/mL (1272.37 μM) (**Figure 3**, **Supplementary Figure S1**, and **Supplementary Table S2**). The results of checkerboard test (**Supplementary Table S2**) and growing curve (**Figure 3** and **Supplementary Figure S1**) suggest that the combination of the compounds with methicillin displays synergistic or partial synergistic effect against MRSA, since the FIC index is 0.5 for **5** with methicillin, and FIC index is 1 for other compounds with methicillin (Sung and Lee, 2008).

### Effect of Compounds 1-5 on Cell Elongation and Cell Membrane

**FIGURE 4:**
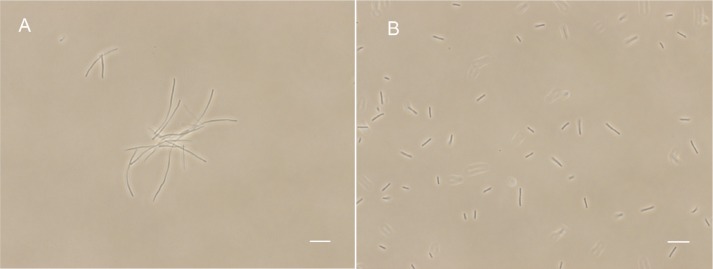
The effect of **5** on *B. subtilis* 168 morphology. Cells of *B. subtilis* 168 were grown with 0.25 μg/mL of compound **5** **(A)**, and without compound **(B)**. The scale bar is 10 μm.

FtsZ targeting compounds can disturb FtsZ function and lead to bacterial cell division inhibition. In order to further investigate the antibacterial mechanism of our compounds, we observed the cell morphological effect of the compounds against *B. subtilis* 168 and *E. coli* 25922 cells. The results show that compounds **1**–**5** can both significantly elongate *B. subtilis* and *E. coli* 25922 cells compared to the control (**Figure 4** and **Supplementary Figures S2**, **S3**). For example, the normal cell length of *B. subtilis* cells is around 5–10 μm (**Figure 4B**). After incubation with **5** at the MIC concentration, the cell length increased was longer than 20 μm (**Figure 4A**). A similar situation happened to *E. coli* 25922, the normal cell length is only 2–4 μm while the treated cells are longer than 12 μm. The cell length increased about 4–6 times compared to the control cells. These phenomena suggest that the benzofuroquinolinium derivatives inhibit the growth of bacteria through a mechanism of cell division inhibition. The histogram of cell length refers to the **Supplementary Figure S4**. Since perturbation of membrane structure can also lead to bacterial cell death, the effect of benzofuroquinolinium derivatives on the bacterial cell membrane was further investigated using a red fluorescent dye FM4-64 on *B. subtilis* cells. In spite of an increased length of *B. subtilis* cells (**Supplementary Figure S5**), these compounds did not induce any detectable perturbation of the cell membrane, as compared to untreated cells (**Supplementary Figure S5**). These results suggest that these benzofuroquinolinium derivatives inhibit bacterial proliferation by inducing cell elongation without perturbing the bacterial membrane.

### Visualization of Z-ring in Bacterial Cells

**FIGURE 5:**
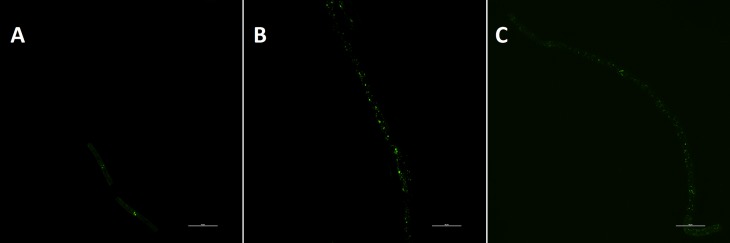
The visualization of Z-ring in bacterial cells. Cells of *B. subtilis* 168 were respectively grown with 1% DMSO **(A)**, 1 μg/mL of compound **2** **(B)** and 0.25 μg/mL of compound **5 (C)**. The scale bar is 10 μm.

Previous studies have shown that some FtsZ inhibitors can perturb FtsZ function to inhibit the formation of Z-ring (Domadia et al., 2007; Haydon et al., 2008; Keffer et al., 2013). The results show that there is a green fluorescent band at the middle of the cell and it becomes some green points at the present of test compounds (**Figure 5**). Both compound **2** and **5** can affect the Z-ring assembly to inhibit cell division and result in cell elongation, probably indicating FtsZ is a target of the benzofuroquinolinium derivatives.

### Effect of Compounds **1–5** on GTPase Activity of FtsZ

**FIGURE 6:**
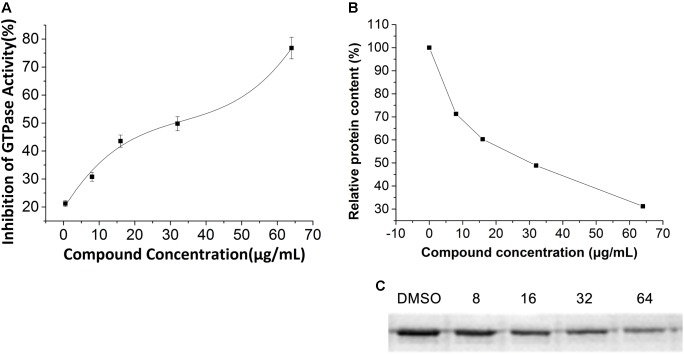
Effect of **5** on polymerization and GTPase Activity of *E. coli* FtsZ. **(A)** Protein were treated with compound **5** at a series concentration of 1, 8, 16, 32, 64 μg/mL. **(B)** Relative protein content of the pellet of the polymerization assay. Optical density analysis relative protein precipitation results by Image J**. (C)** SDS-PAGE of protein pellet after treated with vehicle (5% DMSO) or 8, 16, 32, 64 μg/mL compound **5**. The concentration of protein is 0.4 mg/mL.

To study the interaction between FtsZ and the compounds, *E. coli* FtsZ was cloned, overexpressed, and purified as previous report (Li and Ma, 2015). A GTPase activity assay kit was used to assess the effects of our compounds on the GTPase activity of *E. coli* FtsZ protein. The results showed that all compounds inhibited the GTPase activity of FtsZ protein in a dose dependent manner (**Figure 6A** and **Supplementary Figure S6**). Among the compounds tested for GTPase inhibitory efficacy comparison, **5** is the most effective one. Typically, **5** at a concentration of 64 μg/mL was able to achieve 70% inhibition while other compounds were showing 60% inhibition or less.

### Inhibition of Polymerization of FtsZ With Compound 5

The influence of **5** on the polymerization of the protein was visualized by 12% SDS-PAGE. As shown in **Figures 6B,C**, the precipitation of *E. coli* FtsZ decreased when increasing the concentration of **5**, meaning that the inhibition of protein polymerization is in a dose dependent manner.

### Drug Resistance Study

**FIGURE 7:**
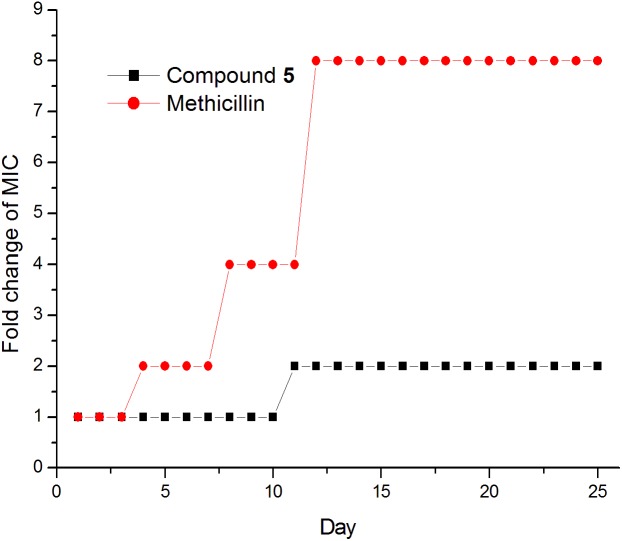
Drug resistance study of compound **1-5.**

The results of compounds **1–5** on drug resistance study suggested that these compounds have a low spontaneous frequency of resistance on *S. aureus* ATCC 29213. As shown in **Figure 7**, *S. aureus* ATCC 29213 did not produce resistant mutants at sublethal concentrations of **5** over a period of 25 days, and the MIC values of **5** did not increase more than 4-fold after 25 passages. Similar results can be found on the compounds **1–4** (data no shown). By contrast, the MIC value of Methicillin against *S. aureus* ATCC 29213 increased 8-fold after 11 passages, indicating the development of resistant mutants. These results indicate that compound **1–5** can kill bacteria effectively and avoid the development of drug resistance. So far, no drug-resistant mutants have been reported in the literature about the FtsZ inhibitors which targeting the GTP binding site, such as chrysophaentin A and pyrimidine derivatives (Plaza et al., 2010; Chan et al., 2017). The possible reason may be due to the importance for recognizing the GTP molecules of this binding site. Mutation of amino acid at this binding site may lead to improper recognition of GTP and hamper normal FtsZ polymerization. Based on these results, we therefore select GTP binding site to predict the possible binding poses of the compounds in the FtsZ in the next test.

### Predictive Binding Mode of Compound 5 in FtsZ

**FIGURE 8:**
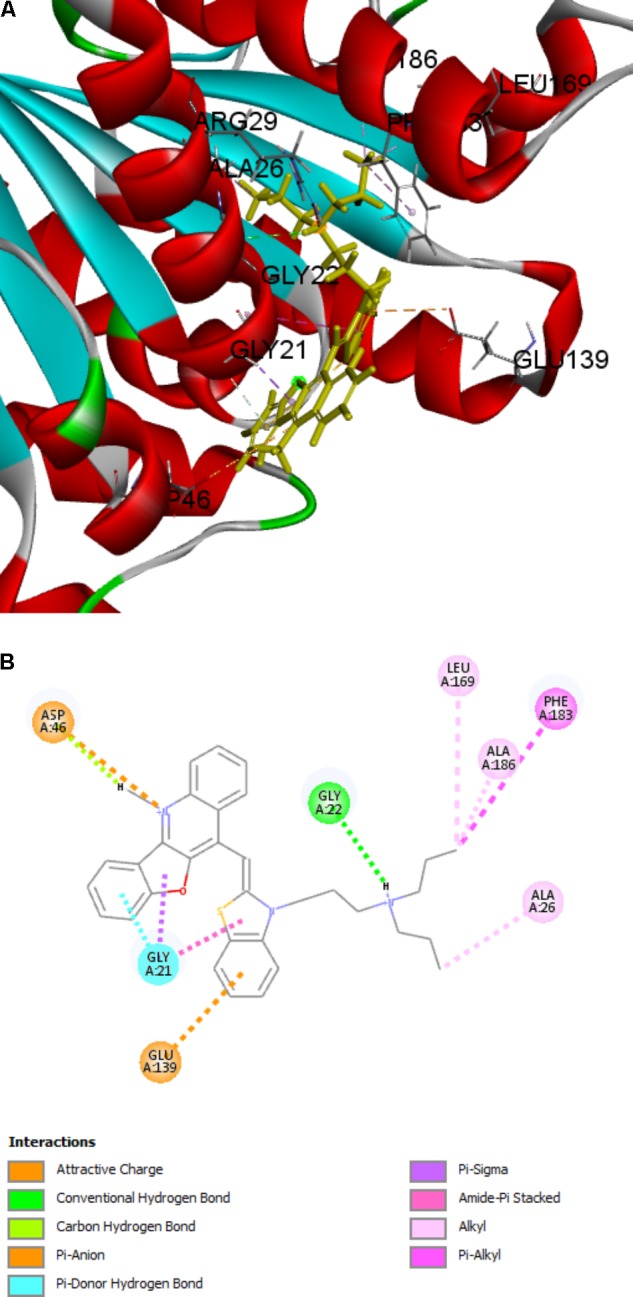
Predicted binding modes of compound **5** bound to FtsZ. **(A)** Compound **5** bound to the GTP-binding site of FtsZ (PDB: 4DXD); **(B)** Predicted interaction between compound **5** and amino acids of FtsZ.

There are two binding site in the FtsZ protein. One is located in the C-terminal interdomain cleft. Most of FtsZ inhibitors, such as PC 190723 and benzamides derivatives, which were found to stablize FtsZ polymerization, are reported to interact with FtsZ via binding into this area. Another pocket is GTP-binding site, compounds predicted to bind into this pocket were found to inhibit the GTPase activity and FtsZ polymerization. Since the features are in accordance with our benzofuroquinolinium derivatives, we carried out the molecular modeling studyusing GTP-binding site of FtsZ. The docking process generated several docking poses. The results revealed that compounds **4** and **5** which containing linear terminal amine group on their side chains have higher scores than that of compounds containing cyclic amine group (**Supplementary Table S4**). On the other hand, the top-score docking poses of **4** and **5** revealed that **5** can found more interactions with FtsZ protein (**Figure 8** and **Supplementary Figure S7**), which are in accordance with the biological actives of these compounds. The top-score docking pose indicates that **5** binds to the FtsZ via interacting with several residues around the GTP-binding pocket (**Figure 8**). The figure shows that there are 8 amino acids involved in the main interactions with **5**. Ala 26, Leu 169, Phe 183 and Ala 186 of FtsZ protein are interact with dipropyl group of **5** through alkyl or Pi-alkyl hydrophobic interactions. Gly 22 can found a conventional hydrogen bond with dipropylamine group of **5**. In addition, Gly 21 and Asp 46 can found non-classical hydrogen bonds with benzofuroquinolinium core of **5**. Moreover, Gly 21 can also interact with **5** through Pi-sigma and amide-Pi stacked interactions. On the other hand, the cationic quinolinium of **5** is predicted to participate in the charge interaction with the negative charged side chain of Asp 46. Last but not least, Glu 139 can interact with the benzene ring of **5** through a Pi-anion interaction (**Figure 8B**).

### Cytotoxicity of Compounds **1–5**

Since FtsZ is a homologue of eukaryotic tubulin, we further tested the toxicity of the compounds to mammalian cells. Two types of normal cells (L929 and HK-2) and one cancer cell line (HepG2) were examined and the inhibition rates are shown in **Table 2**. We treated the cells with 50 μg/mL (>20-fold of MIC against MRSA) of our compounds and found that the cell inhibition rates are about 50–65%, indicating that compounds may have no significant cytotoxicity on mammalian cells.

**Table 2:** Inhibitory effects of compounds on mammalian cells.

Compound	HK2 (%)	L929 (%)	HepG2 (%)
concentration			
(50 μg/mL)			
**1**	53	61	64
**2**	65	66	64
**3**	53	62	61
**4**	53	61	57
**5**	65	54	61


*The growth inhibitory rate = (A_*control*_ – A_*sample*_)/A_*control*_, A is the OD of wells. nd for not detected.*

### Hemolytic Activity

**FIGURE 9:**
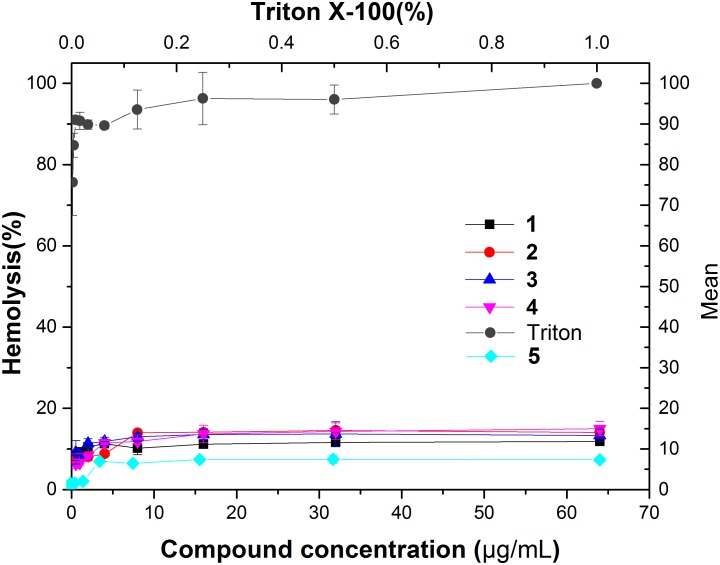
Hemolytic activity of compound **1–5**. Human erythrocytes were treated with serial dilutions of Triton X-100 (0.002–1%) or compounds **1–5** (0.125–64 g/ml).

To investigate the toxicity of the compounds to human erythrocytes, we tested the hemolytic activity of compounds **1–5** using a human red cell lysis assay. The results showed that red blood cells treated with the detergent Triton X-100 were almost completely hemolyzed. On the other hand, 64 μg/mL of our compounds did not show significant hemolysis (HC_50_ > 64 μg/mL) (**Figure 9**). When compared with antibacterial activity of these compounds against MRSA, all the selectivity indices (HC_50_/MIC) are higher than 32 (**Table 3**), suggesting that our compounds do not have toxicity to human erythrocytes.

**Table 3:** Selectivity of compounds **1–5.**

Compound	MIC (μg/mL)	HC_50_ (μg/mL)	Selectivity
	MRSA	Human	(HC_50_/MIC)
	BAA-41	erythrocytes	
**1**	1	>64	>64
**2**	2	>64	>32
**3**	1	>64	>64
**4**	2	>64	>32
**5**	1	>64	>64


*HC_*50*_ value is the concentration required to lyse 50% of human red blood cells.*

## Discussion

From the above studies, compounds **1–5** showed different degree of potency and a broad spectrum antibacterial activity. The bacterial experiments showed that the compounds restored the antibacterial activity of methicillin and elongate the *B. subtilis* 168 by inhibiting the cell division process. In the previous study, some FtsZ inhibitors such as quinoline derivatives, benzamide derivatives and 9-phenoxyalkylberberine derivatives also gave similar cell elongation effects. These phenomena encouraged us to further investigate the mode of action of benzofuroquinolinium derivatives targeting FtsZ. It was found that these compounds can inhibit the polymerization and decreased the GTPase activity of FtsZ in vitro. Moreover, the cytotoxicity data of **1–5** against two normal mammalian cells revealed that these compounds were hypotoxicity.

Among these tested compounds, **5** shows remarkable inhibition against Gram-positive strains (including MRSA and VRE) with MIC values from 0.25–4 μg/mL (0.48–7.65 μM). It is as good as or even better than some reported FtsZ inhibitors with high potency. For example, the MIC of 9-phenoxyalkylberberine derivative **2** against MRSA and VREF is from 2 to 8 μg/mL (Sun et al., 2014). The cinnamaldehyde derivatives exhibited MIC values of 4 to 64 μg/mL against MRSA (Li and Ma, 2015). Besides, **5** can strongly inhibit the Gram-negative strains with the MIC values from 1 to 8 μg/mL (1.91–15.30 μM). On the other hand, compounds 9-phenoxyalkylberberine derivative **2** and quinuclidine **1** were reported as FtsZ inhibitors with the MIC value of 32 μg/mL against *E. coli.*, and cinnamaldehyde derivatives inhibited the growth of *E. coli* with MIC value of 128 μg/mL (Li and Ma, 2015). The antibacterial activities of **5** is much stronger compared to 9-phenoxyalkylberberine derivatives, quinuclidine derivative, and cinamaldehyde derivatives, suggesting that **5** possesses a better ability to pass the outer membrane of Gram-negative bacteria. On the other hand, it was reported that GTPase activity of FtsZ can affect its assembly and most of the FtsZ inhibitors can restraint the GTPase activity (Margalit et al., 2004; Margolin, 2005; Chan et al., 2013; Sun et al., 2014; Hurley et al., 2016). Compound **5** can inhibit 50% inhibition of GTP hydrolysis at the concentration of 32 μg/mL (∼61.22 μM). From the above biological assays, **5** is targeting FtsZ with a strong antibacterial activity.

## Conclusion

In conclusion, we demonstrated that the class of benzofuroquinolinium derivatives exhibit potent antibacterial activities against Gram-positive, Gram-negative, and even drug resistant bacteria. Furthermore, the compounds can restore the antibacterial activity of methicillin and perturb the Z-ring assembly. The mode of action of the compound was also studied through biochemical evaluations. The results indicate that compound **5** can effectively inhibit the polymerization and GTPase activity of FtsZ in a dose-dependent manner. In addition, the compound probably interacts with the GTP-binding site and disrupts the GTPase activity and polymerization of FtsZ, which cause abnormal bacterial cell division resulting cell death. More importantly, the benzothiazole-substituted benzofuroquinolinium derivatives were shwoing low cytotoxicity on mammalian cells. Therefore, the class of compounds could be developed as the potent antibacterial agents against drug resistant bacteria.

## Author Contributions

NS, YJ-L, and KY-W conceived and designed the experiments. NS, Y-YZ, RL-D, SY-C, and Z-YF performed the experiments. NS, Y-JL, Y-YZ, and R-LD analyzed the data. L-YS, TL, and Z-HL contributed reagents, materials, and analysis tools. NS, Y-JL, and K-YW wrote the paper.

## Conflict of Interest Statement

The authors declare that the research was conducted in the absence of any commercial or financial relationships that could be construed as a potential conflict of interest.

## Footnotes

1 http://www.rcsb.org
