# Supplementary material for: Study of Benzofuroquinolinium Derivatives as a New Class of Potent Antibacterial Agent and the Mode of Inhibition Targeting FtsZ
Source: Front Microbiol. 2018 Aug 17;9:1937. doi: 10.3389/fmicb.2018.01937 (PMC6107709; doi:10.3389/fmicb.2018.01937)
Supplement: Supplementary file 1 [file Data_Sheet_1.docx]

Supplementary Material

**List of contents:**

1. Minimum Inhibitory Concentrations of Compounds **1**-**5** (molar concentration)
2. Synergistic effect of compounds **1**-**5** with Methicillin
3. Visualization of bacterial morphology and cell membrane
4. GTPase Activity Assay
5. Molecular modeling
6. Synthesis route and characterization enzothiazole-substituted benzofuroquinolinium derivatives
7. References
8. Minimum Inhibitory Concentrations of Compounds 1-5 (molar concentration)

Table S1. Minimum inhibitory concentrations of compounds 1-5 against a series of bacterial strains.

| Organism | MIC (μM) | | | | | |
| --- | --- | --- | --- | --- | --- | --- |
|  | **1** | **2** | **3** | **4** | **5** | Methicillin |
| (+)*B. subtilis* 168 | 0.98 | 2.03 | 0.99 | 0.96 | 0.48 | <2.49 |
| (+)*S. aureus* ATCC 29213 | 7.86 | 8.12 | 7.89 | 3.84 | 0.48 | <2.49 |
| (+)*S. aureus* ATCC 25923 | 3.93 | 4.06 | 3.95 | 3.84 | 1.91 | <2.49 |
| (+)*S. aureus* ATCC 43300^a^ | 7.86 | 8.12 | 7.89 | 7.68 | 1.91 | 1272.37 |
| (+)*S. aureus* ATCC BAA41^a^ | 1.97 | 4.06 | 1.97 | 1.92 | 1.91 | 2544.75 |
| (+)*S. aureus* ATCC 33591^a^ | 15.73 | 8.12 | 7.89 | 7.68 | 1.91 | 2544.75 |
| (+)*S. aureus* ATCC 33592^a^ | 7.86 | 8.12 | 3.95 | 3.84 | 0.96 | 1272.37 |
| (+)*S. aureus* ATCC BAA-1720^a^ | 3.93 | 8.12 | 3.95 | 3.84 | 1.91 | 2544.75 |
| (+)*E. faecalis* ATCC 29212 | 31.46 | 16.24 | 15.79 | 15.36 | 7.65 | 2.49 |
| (+)*E. faecalis* ATCC 700221 | 7.86 | 16.24 | nd | 3.84 | 1.91 | 3.73 |
| (-)*E.coli* ATCC 25922 | 15.73 | 8.12 | 3.95 | 3.84 | 1.91 | 7.46 |
| (-)*E.coli* ATCC BAA-2469 | 15.73 | 8.12 | 3.95 | 7.68 | 1.91 | >159.05 |
| (-)*A. Baumannii* ATCC 19606 | 62.91 | 32.48 | 31.58 | 30.73 | 15.30 | >159.05 |
| (-)*P. aeruginosa* ATCC BAA-2108 | 31.46 | 32.48 | 31.58 | 30.73 | 7.65 | >159.05 |
| (-)*K. pneumoniae* ATCC BAA-2470 | 125.82 | 32.48 | 31.58 | 30.73 | 15.30 | >159.05 |

^a^ These strains are MRSA. (+) for Gram-positive and (-) for Gram-negative. nd for not detected.

1. Synergistic effect of compounds 1-5 with Methicillin

The results of compounds **1-5** combined with Methicillin against *S. aureus* ATCC BAA41 (MRSA) have shown at Figure S1.


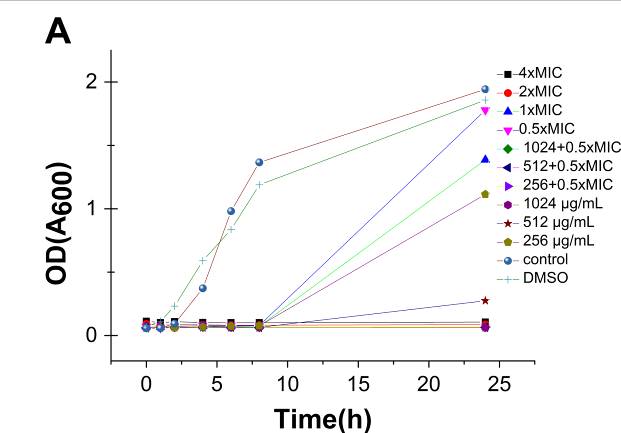

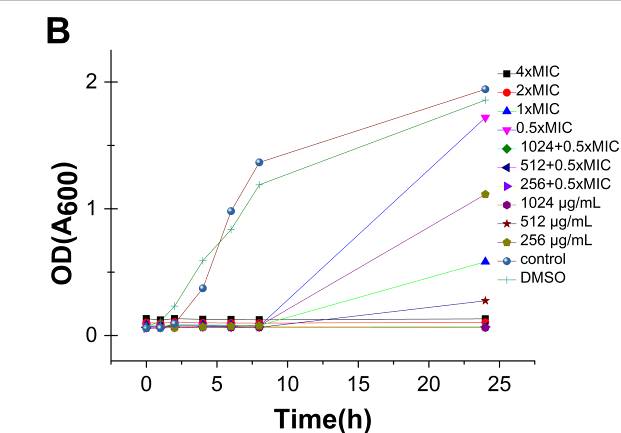

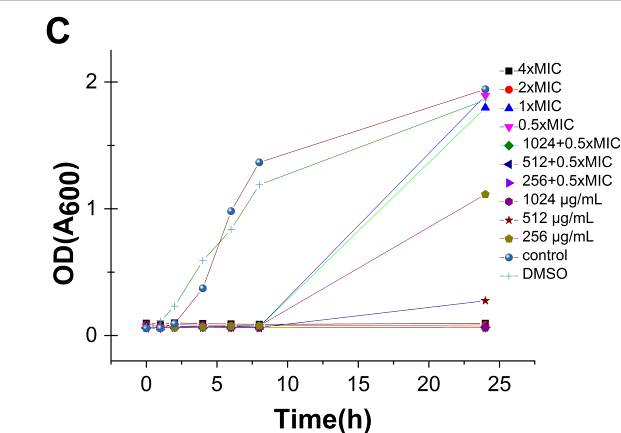

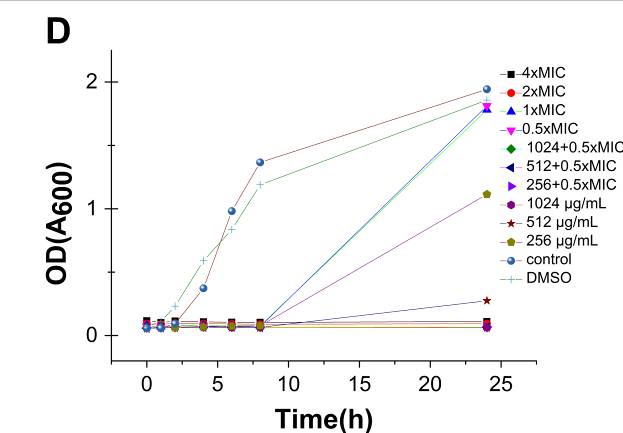

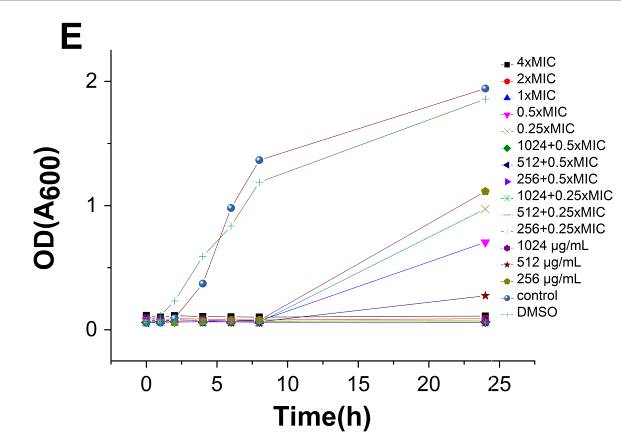


**Figure S1. Effects of benzofuroquinolinium derivatives combination with methicillin on *S. aureus* ATCCBAA41.**

*S. aureus* ATCCBAA41 was treated with methicillin and compounds **1-5** in different combinations of concentrations. (A) Methicillin with compound **1** (B) Methicillin with compound **2** (C) Methicillin with compound **3** (D) Methicillin with compounds **4** (E) Methicillin with compounds **5**.

Table S2. MIC values (in μg/mL) of compounds 1-5 combined with methicillin (MET) against MRSA ATCC BAA-41.

| Single compound MIC | | | | | |
| --- | --- | --- | --- | --- | --- |
| MET | 1 | 2 | 3 | 4 | 5 |
| 1024 | 1 | 2 | 1 | 2 | 1 |
| Combination of compounds 1-5 and methicillin MIC | | | | | |
|  | MET+1 | MET+2 | MET+3 | MET+4 | MET+5 |
|  | 256+0.5 | 256+1 | 256+0.5 | 256+1 | 256+0.25 |
| FIC index | 0.75 | 0.75 | 0.75 | 0.75 | 0.5 |

1. Visualization of bacterial morphology and cell membrane

The effect of test compounds on bacterial morphology have shown below. (Figure S2-S4)


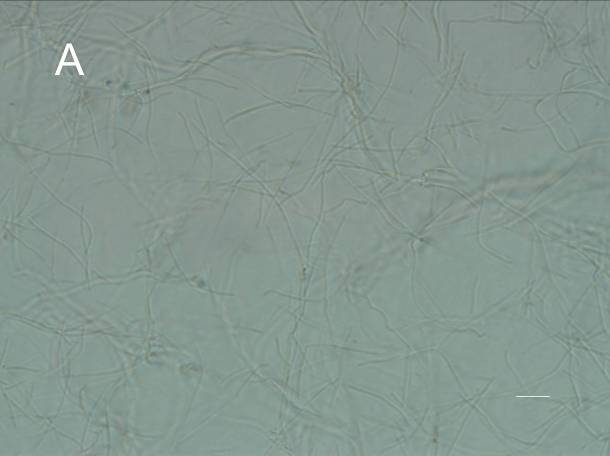

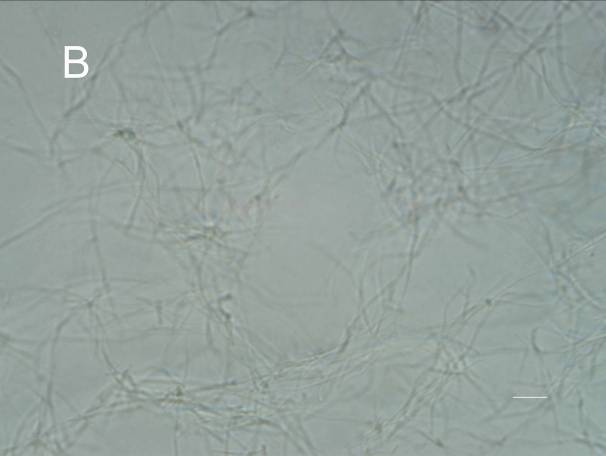

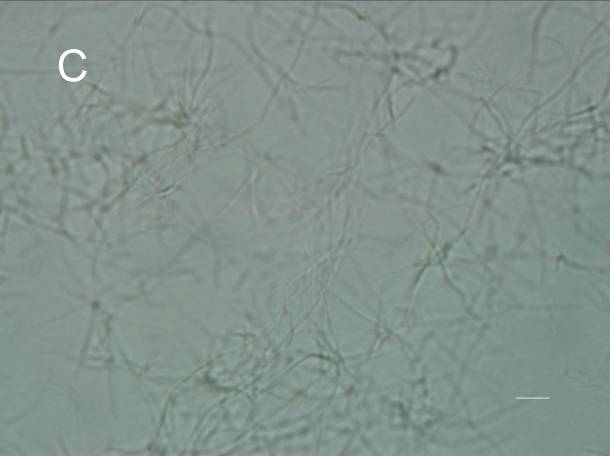

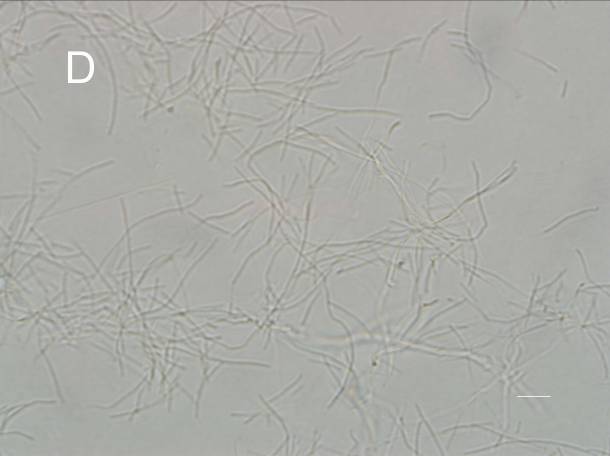


**Figure S2. The effect of benzofuroquinolinium derivatives on *B. subtilis* 168 morphology.** **(A)** *B. subtilis* 168 cells with compound **1** at 1 μg/mL, **(B) 2** at 2 μg/mL, **(C) 3** at 1 μg/mL and **(D) 4** at 0.5 μg/mL. The scale bar is 10 μm.


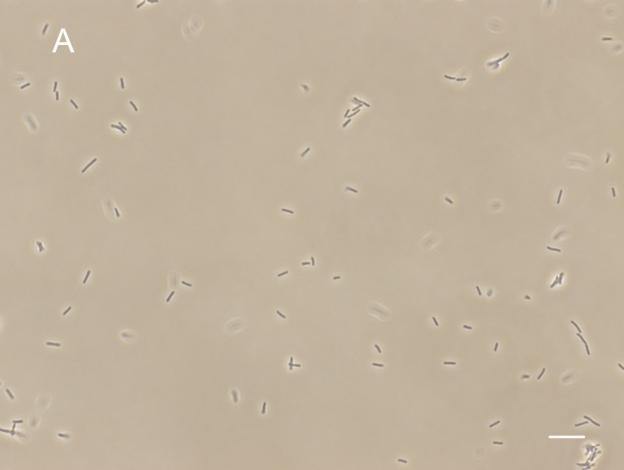

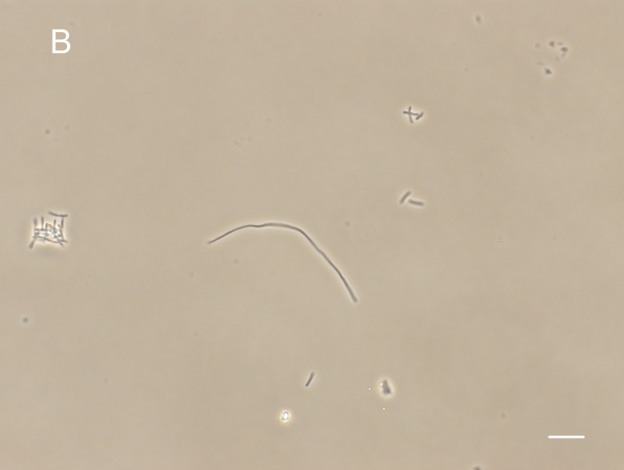

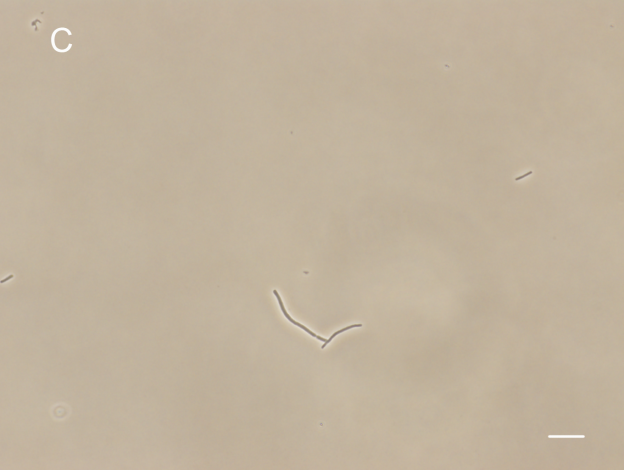

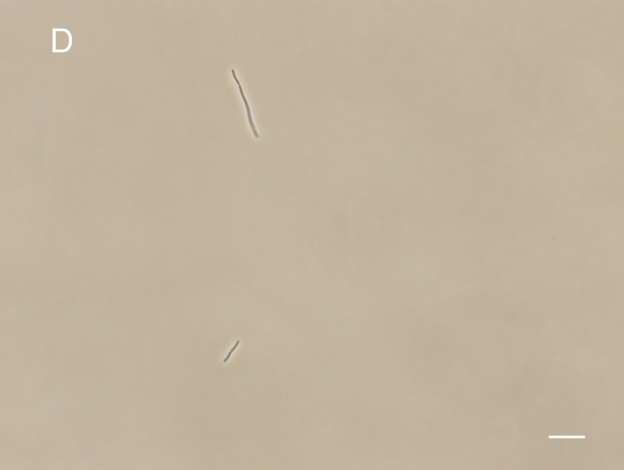

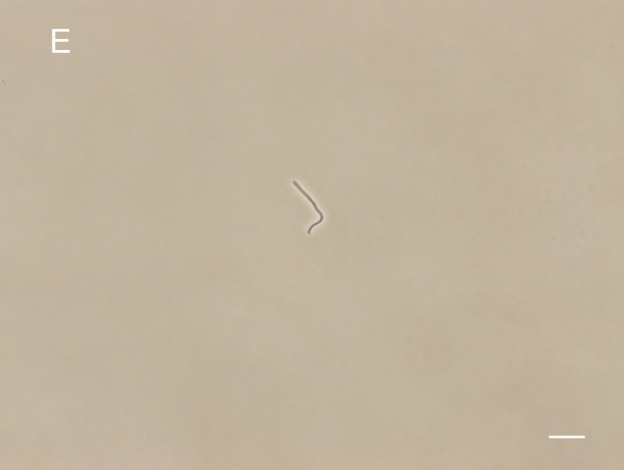


**Figure S3. The effect of benzofuroquinolinium derivatives on *E. coli* 25922 morphology.** *E. coli* 25922 cells with **(A)** 1% DMSO, **(B) 1** at 4 μg/mL, **(C) 2** at 2 μg/mL, **(D) 4** at 2 μg/mL and **(E)** **5** at 0.5 μg/mL. The scale bar is 10 μm.







**Figure S4. The histograms of cell length distribution.** The histograms show average and standard error from three independent experiments.

**
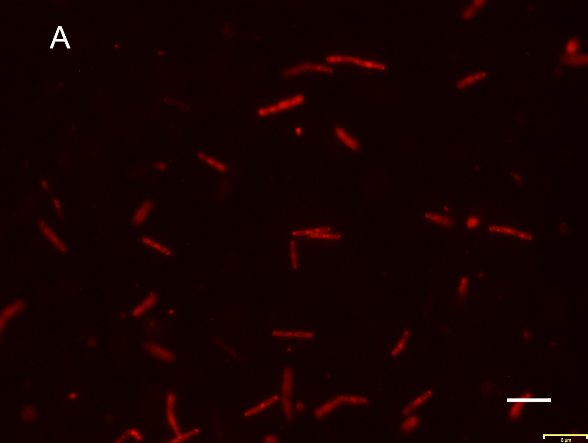

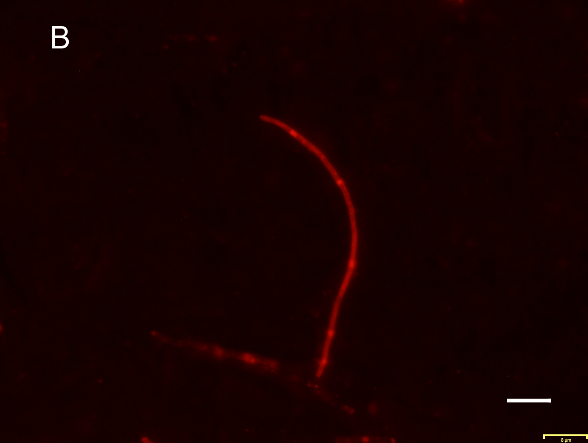
**


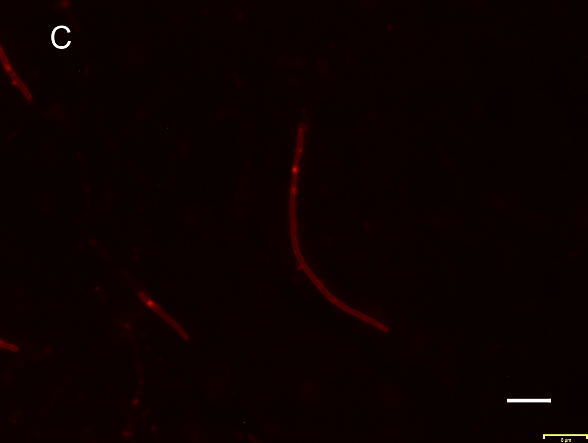

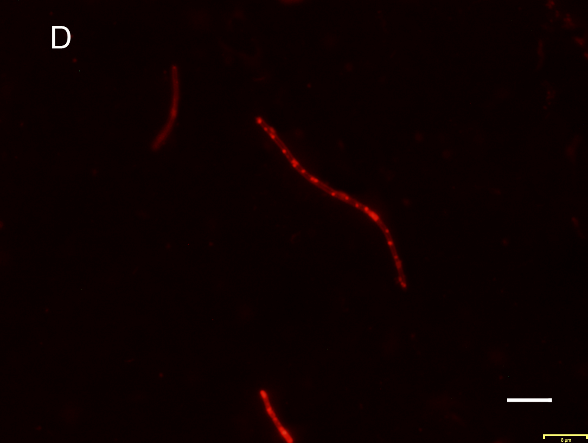


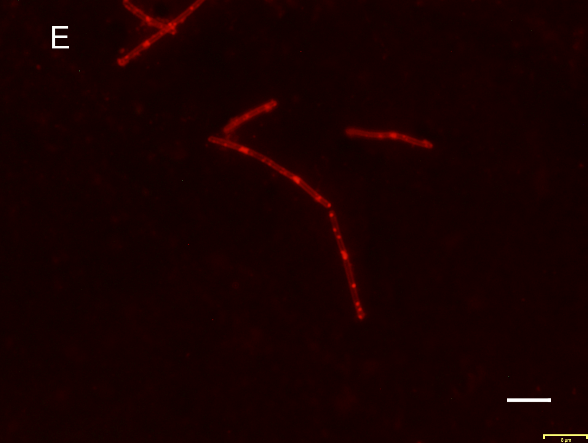

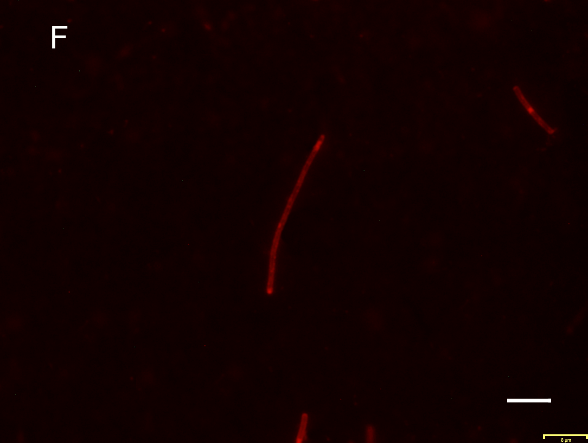


**Figure S5. Effect of benzofuroquinolinium derivatives 1-5 on the cell membrane of *B. Subtilis* 168.** Cells with **(A)** 1% DMSO, **(B) 1** at 1 μg/mL, **(C) 2** at 2 μg/mL, **(D) 3** at 1 μg/mL, **(E)** **4** at 0.5 μg/mL and **(F)** **5** at 0.5 μg/mL. The scale bar is 8 μm.

1. GTPase Activity Assay

The Figure S6 shows the results of compounds **1-4** affects GTPase activity of FtsZ. The phosphorus standard curve has been measured and shown at Table 2, the standard curve equation is y = 0.0092x + 0.289 and R^2^= 0.996.

**Table S3** Phosphorus standard curve.

| Pi-standard concentration（μM） | 0 | 12.5 | 25 | 31.25 | 37.5 | 50 |
| --- | --- | --- | --- | --- | --- | --- |
| OD_620_ | 0.1991 | 0.3273 | 0.4546 | 0.493 | 0.5641 | 0.6597 |


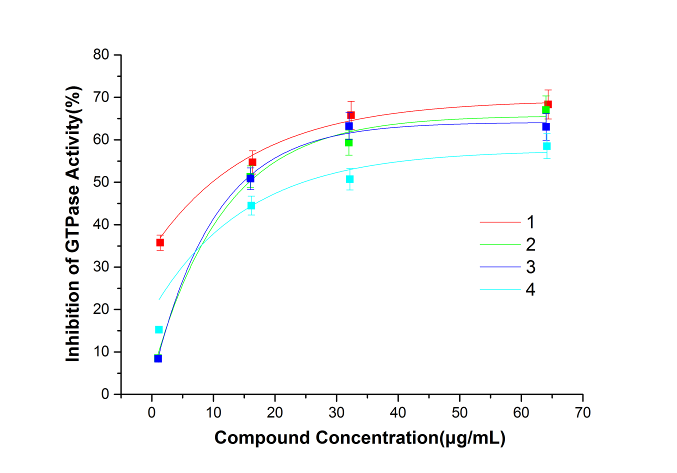


**Figure S6. Effect of Compounds on GTPase Activity of *E. coli* FtsZ.** Protein were treated with compounds **1**-**4** at a series concentration of 1, 16, 32, 64 μg/mL.

1. Molecular modeling

The Table S4 shows the CDOCKER ENERGY of compounds **1-5** and protein. Comparing the minimum energy values of each compound, we found that the energy of compound 5 was the lowest, indicating that compound 5 is more prone to protein binding than the other compounds. The Figure S7 shows the top-score docking pose and 2D interaction diagram of compound **4** with protein.

**Table S4** CDOCKER data of compounds **1-5** and protein.

| Compound | Number of poses | CDOCKER ENERGY | |
| --- | --- | --- | --- |
|  |  | Maximum | Minimum |
| **1** | 10 | -21.570 | -22.4486 |
| **2** | 10 | -29.809 | -35.6589 |
| **3** | 20 | -21.4118 | -29.1182 |
| **4** | 10 | -29.2475 | -38.2664 |
| **5** | 8 | -25.5836 | -40.9953 |


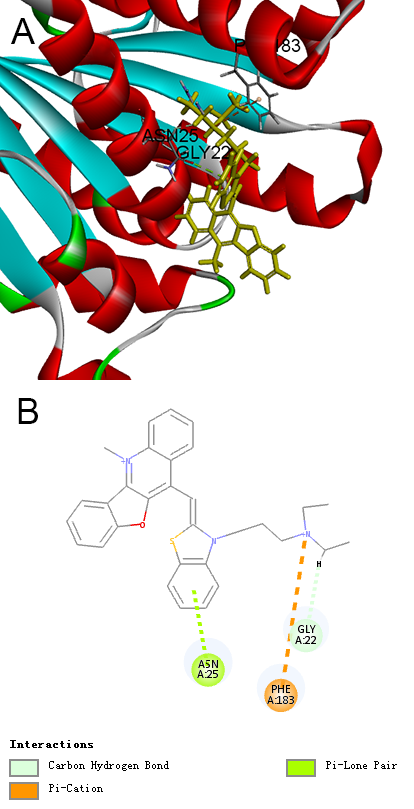


**Figure S7. Predicted binding modes of compound 4 bound to FtsZ.**

(A) compound **4** bound to the GTP-binding site of FtsZ (PDB: 4DXD); (B) Predicted interaction between compound **4** and amino acids of FtsZ.

1. Synthesis of benzothiazole-substituted benzofuroquinolinium derivatives

The test compounds were synthesized according to the previous report (Lu et al., 2015) The target compounds were confirmed by NMR and HRMS.

*(5-Methyl-11-((3-(3-morphoine)-propylbenzo[d]thiazol-2(3H)-ylidene)methyl)benzofuro[3,2-b]quinolin-5-ium iodide (****1****)*

Purple solid; mp 213~215^o^C;^1^H NMR (400 MHz, DMSO-*d_6_*):δ 8.78 (d, *J* = 8.2 Hz, 1H), 8.69 (d, *J* = 8.0 Hz, 1H), 8.43 (d, *J* = 8.2 Hz, 1H), 8.13~8.05 (m, 1H), 8.02 (s, 1H), 7.95 (d, *J* = 7.3 Hz, 2H), 7.79 (d, *J* = 8.5 Hz, 2H), 7.69 (s, 1H), 7.58 (s, 1H), 7.39 (d, *J* = 7.6 Hz, 1H), 6.95 (s, 1H), 4.66 (s, 5H), 3.46 (s, 4H), 2.48~ 2.39 (m, 2H), 2.28 (s, 4H), 2.09 (s, 2H).^13^C NMR (100 MHz, DMSO-*d_6_*):δ 160.92 (s), 156.46 (s), 141.37 (s), 139.47 (s), 137.84 (s), 136.35 (s), 134.48 (s), 133.46 (s), 132.99 (s), 128.01 (s), 126.95 (s), 126.33 (s), 125.76 (s), 125.40 (s), 125.34 (s), 124.68 (s), 123.10 (s), 122.95 (s), 118.32 (s), 117.99 (s), 113.71 (s), 113.43 (s), 84.94 (s), 63.85 (s), 55.49 (s), 53.75 (s), 44.80 (s), 38.64 (s), 23.63 (s). ESI-MS: m/z = 508 [M -I]^+^. HRMS (ESI): m/z calcd for C_31_H_29_N_3_O_2_S ([M -I]^+^) 508.2053; found 508.2055.

*5-Methyl-11-((3-(3-pyrrolidine)-propylbenzo[d]thiazol-2(3H)-ylidene)methyl)benzofuro[3,2-b]quinolin-5-ium iodide(****2****)*

Purple solid; mp 226~228^o^C; ^1^H NMR (400 MHz, DMSO-*d_6_*):δ 8.82 (d, *J* = 8.3 Hz, 1H), 8.73 (d, *J* = 8.2 Hz, 1H), 8.49 (d, *J* = 8.9 Hz, 1H), 8.12 (t, *J* = 7.8 Hz, 1H), 8.05 (d, *J* = 8.4 Hz, 1H), 7.97 (dd, *J* = 14.5, 7.5 Hz, 2H), 7.87 (t, *J* = 7.6 Hz, 1H), 7.78 (d, *J* = 8.2 Hz, 1H), 7.72 (t, *J* = 7.6 Hz, 1H), 7.59 (t, *J* = 7.7 Hz, 1H), 7.38 (t, *J* = 7.6 Hz, 1H), 6.95 (s, 1H), 4.85~4.53 (m, 5H), 3.57 (d, *J* = 25.6 Hz, 2H), 3.49 (s, 2H), 3.10 (s, 2H), 2.27 (s, 2H), 2.04 (s, 2H), 1.87 (d, *J* = 5.0 Hz, 2H). ^13^C NMR (100 MHz, DMSO-*d_6_*): δ160.35 (s), 156.73 (s), 140.85 (s), 139.90 (s), 137.81 (s), 137.10 (s), 134.82 (s), 133.88 (s), 133.17 (s), 128.11 (s), 127.34 (s), 126.62 (s), 125.67 (s), 125.57 (s), 125.48 (s), 124.72 (s), 123.41 (s), 123.12 (s), 118.50 (s), 117.99 (s), 113.80 (s), 112.92 (s), 84.37 (s), 54.00 (s), 51.69 (s), 43.43 (s), 38.93 (s), 23.98 (s), 23.16 (s). ESI-MS: m/z = 492 [M -I]^+^. HRMS (ESI): m/z calcd for C_31_H_29_N_3_OS ([M - I]^+^) 492.2104; found 492.2111.

*5-Methyl-11-((3-(3-piperidine)-propylbenzo[d]thiazol-2(3H)-ylidene)methyl)benzofuro[3,2-b]quinolin-5-ium iodide(****3****)*

Purple solid; mp 210~213^o^C; ^1^H NMR (400 MHz, DMSO-*d_6_*):δ 8.83 (d, *J* = 8.4 Hz, 1H), 8.72 (d, *J* = 8.2 Hz, 1H), 8.49 (d, *J* = 8.9 Hz, 1H), 8.12 (t, *J* = 7.8 Hz, 1H), 8.04 (t, *J* = 11.3 Hz, 1H), 7.97 (dd, *J* = 12.9, 7.6 Hz, 2H), 7.86 (t, *J* = 7.6 Hz, 1H), 7.78 (d, *J* = 8.3 Hz, 1H), 7.71 (t, *J* = 7.6 Hz, 1H), 7.59 (t, *J* = 7.7 Hz, 1H), 7.39 (t, *J* = 7.6 Hz, 1H), 6.95 (s, 1H), 4.89~4.51 (m, 5H), 3.49 (d, *J* = 11.6 Hz, 2H), 3.40 (s, 2H), 2.96 (dd, *J* = 21.1, 9.9 Hz, 2H), 2.30 (s, 2H), 1.83 (d, *J* = 14.0 Hz, 2H), 1.74~1.36 (m, 4H). ^13^C NMR (100 MHz, DMSO-*d_6_*): δ 160.40 (s), 156.71 (s), 140.85 (s), 139.87 (s), 137.80 (s), 137.07 (s), 134.81 (s), 133.87 (s), 133.17 (s), 128.10 (s), 127.35 (s), 126.62 (s), 125.66 (s), 125.59 (s), 125.48 (s), 124.73 (s), 123.40 (s), 123.13 (s), 118.49 (s), 117.98 (s), 113.79 (s), 112.96 (s), 84.44 (s), 53.42 (s), 52.80 (s), 43.46 – 43.36 (m), 38.93 (s), 23.09 (s), 22.18 (s), 21.61 (s). ESI-MS: m/z = 506 [M -I]^+^.HRMS (ESI): m/z calcd for C_32_H_31_N_3_OS ([M - I]^+^) 506.2267; found 506.2267.

*5-Methyl-11-((3-(3-diethylamine)-propylbenzo[d]thiazol-2(3H)-ylidene)methyl)benzofuro[3,2-b]quinolin-5-ium iodide(****4****)*

Purple solid; mp 228~230^o^C; ^1^H NMR (400 MHz, DMSO-*d_6_*):δ 8.80 (d, *J* = 8.7 Hz, 1H), 8.72 (d, *J* = 8.1 Hz, 1H), 8.49 (d, *J* = 8.9 Hz, 1H), 8.15 – 8.08 (m, 2H), 8.04 (d, *J* = 8.4 Hz, 1H), 7.97 (dd, *J* = 16.0, 7.6 Hz, 2H), 7.87 ~7.79 (m, 2H), 7.74~7.69 (m, 1H), 7.59 (t, *J* = 7.7 Hz, 1H), 7.38 (t, *J* = 7.6 Hz, 2H), 6.96 (s, 1H), 4.70 (d, *J* = 8.5 Hz, 5H), 3.43 (s, 2H), 2.24 (s, 4H), 1.24 (t, *J* = 7.1 Hz, 7H), 1.18~1.14 (m, 2H). ^13^C NMR (100 MHz, DMSO-*d_6_*):δ 160.27 (s), 156.72 (s), 140.84 (s), 139.89 (s), 137.81 (s), 137.12 (s), 134.78 (s), 133.89 (s), 133.18 (s), 128.13 (s), 127.36 (s), 126.55 (s), 125.68 (s), 125.55 (s), 125.48 (s), 124.72 (s), 123.41 (s), 123.13 (s), 118.51 (s), 117.99 (s), 113.79 (s), 112.94 (s), 84.49 (s), 47.14 (s), 43.33 (s), 41.86 (s), 38.94 (s), 11.53 (s), 9.21 (s).ESI-MS: m/z = 494 [M -I]^+^. HRMS (ESI): m/z calcd for C_31_H_31_N_3_O_2_S ([M - I]^+^) 494.2261; found 494.2268.

*5-Methyl-11-((3-(3-dipropylamine)-propylbenzo[d]thiazol-2(3H)-ylidene)methyl)benzofuro[3,2-b]quinolin-5-ium iodide(****5****)*

Purple solid; mp 215~217^o^C; ^1^H NMR (400 MHz, DMSO-*d_6_*):δ 9.12 (s, 1H), 8.81 (d, *J* = 7.0 Hz, 1H), 8.73 (d, *J* = 7.7 Hz, 1H), 8.49 (d, *J* = 8.4 Hz, 1H), 8.06 (t, *J* = 22.3 Hz, 2H), 7.96 (s, 2H), 7.79 (d, *J* = 26.8 Hz, 2H), 7.72 (s, 1H), 7.60 (s, 1H), 7.39 (s, 1H), 6.97 (s, 1H), 4.71 (s, 5H), 3.45 (s, 2H), 3.10 (s, 4H), 2.26 (s, 2H), 1.69 (s, 4H), 0.91 (s, 6H). ^13^C NMR (100 MHz, DMSO-*d_6_*):δ 160.30 (s), 156.70 (s), 140.83 (s), 139.85 (s), 137.81 (s), 137.09 (s), 134.72 (s), 133.88 (s), 133.18 (s), 128.14 (s), 127.34 (s), 126.50 (s), 125.68 (s), 125.58 (s), 125.49 (s), 124.73 (s), 123.41 (s), 123.14 (s), 118.51 (s), 117.98 (s), 113.79 (s), 112.98 (s), 84.57 (s), 54.12 (s), 49.16 (s), 43.30 (s), 38.94 (s), 19.52 (s), 17.26 (s), 11.28 (s). ESI-MS: m/z= 522 [M -I]^+^. HRMS (ESI): m/z calcd for C_33_H_35_N_3_OS ([M - I]^+^) 522.2574; found 522.2583.

**
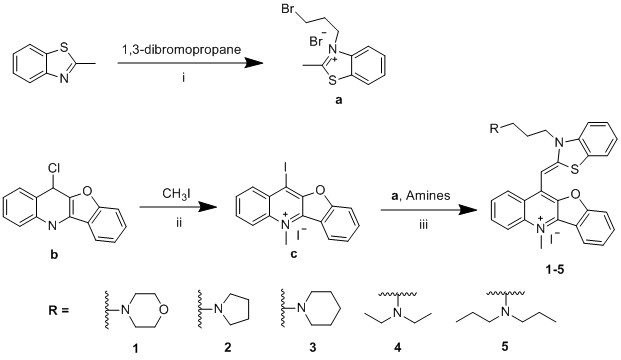
Scheme 1 Synthetic route for compounds 1-5.** Reagents and conditions: (i)1,3-dibromopropane, 110^o^C, 18 h; (ii) tetramethylene sulfone, iodmethane, 68^o^C, 10 h; (iii) methanol, sodium bicarbonate, 1-(3-Bromo)-propyl-2-methylbenzothiazolium bromide, morpholine, pyrrolidine, piperidine, 4-methylpyridine, diethylamine, dipropylamine, 68^o^C, 3 h.

**Reference:**

Liu, I.X., Durham, D.G., and Richards, R.M.E. (2000). Baicalin Synergy with β‐Lactam Antibiotics Against Methicillin‐resistant Staphylococcus aureus and Other β‐Lactam‐resistant Strains of S. aureus. *Journal of Pharmacy and Pharmacology* 52**,** 361-366.

Lu, Y.-J., Wang, Z.-Y., Hu, D.-P., Deng, Q., Huang, B.-H., Fang, Y.-X., Zhang, K., Wong, W.-L., and Chow, C.-F. (2015). Benzothiazole-substituted benzofuroquinolinium dyes as new fluorescent probes for G-quadruplex DNA. *Dyes and Pigments* 122**,** 94-102.

Sun, N., Chan, F.-Y., Lu, Y.-J., Neves, M.A., Lui, H.-K., Wang, Y., Chow, K.-Y., Chan, K.-F., Yan, S.-C., and Leung, Y.-C. (2014). Rational design of berberine-based FtsZ inhibitors with broad-spectrum antibacterial activity. *PloS one* 9**,** e97514.

Sung, W.S., and Lee, D.G. (2008). Mechanism of decreased susceptibility for Gram-negative bacteria and synergistic effect with ampicillin of indole-3-carbinol. *Biological and Pharmaceutical Bulletin* 31**,** 1798-1801.
